# Effect of Plant Derived Antimicrobials on *Salmonella* Enteritidis Adhesion to and Invasion of Primary Chicken Oviduct Epithelial Cells *in vitro* and Virulence Gene Expression

**DOI:** 10.3390/ijms140510608

**Published:** 2013-05-21

**Authors:** Indu Upadhyaya, Abhinav Upadhyay, Anup Kollanoor-Johny, Michael J. Darre, Kumar Venkitanarayanan

**Affiliations:** Department of Animal Science, University of Connecticut, Storrs, CT 06269, USA; E-Mails: indu.upadhyaya@uconn.edu (I.U.); abhinav.upadhyay@uconn.edu (A.U.); kollanooranup@gmail.com (A.K.-J.); michael.darre@uconn.edu (M.J.D.)

**Keywords:** *Salmonella*, plant-derived antimicrobials, oviduct, macrophages, gene expression

## Abstract

*Salmonella* Enteritidis (SE) is a major foodborne pathogen in the United States and one of the most frequently reported *Salmonella* serotypes globally. Eggs are the most common food product associated with SE infections in humans. The pathogen colonizes the intestinal tract in layers, and migrates to reproductive organs systemically. Since adhesion to and invasion of chicken oviduct epithelial cells (COEC) is critical for SE colonization in reproductive tract, reducing these virulence factors could potentially decrease egg yolk contamination. This study investigated the efficacy of sub-inhibitory concentrations of three plant-derived antimicrobials (PDAs), namely carvacrol, thymol and eugenol in reducing SE adhesion to and invasion of COEC, and survival in chicken macrophages. In addition, the effect of PDAs on SE genes critical for oviduct colonization and macrophage survival was determined using real-time quantitative PCR (RT-qPCR). All PDAs significantly reduced SE adhesion to and invasion of COEC (*p* < 0.001). The PDAs, except thymol consistently decreased SE survival in macrophages (*p* < 0.001). RT-qPCR results revealed down-regulation in the expression of genes involved in SE colonization and macrophage survival (*p* < 0.001). The results indicate that PDAs could potentially be used to control SE colonization in chicken reproductive tract; however, *in vivo* studies validating these results are warranted.

## 1. Introduction

Eggs constitute a vital part of the American diet with an annual per capita consumption of approximately 250 eggs [1]. Approximately 90 billion eggs are produced and 67.5 billion shell eggs being consumed annually in the USA [1]. Thus, the microbiological safety of eggs is a major concern from public health and economic perspectives. *Salmonella enterica* serovar Enteritidis (SE) is one of the most common bacterial agents causing enteric disease in humans, largely due to the consumption of contaminated eggs [2–6]. Humans contract SE infection *via* consumption of contaminated, raw or undercooked eggs. In light of increasing evidence linking human salmonellosis with consumption of eggs, the Food and Drug Administration (FDA) in 2009 [7] announced that eggs constitute the primary source of SE infections to humans, and issued a final rule that requires egg producers to implement measures to prevent the pathogen from contaminating eggs on the farm and further growth during storage and transportation [7].

Cecum is the primary site of SE colonization in chicken [8,9], with cecal carriage of the pathogen leading to contamination of ovaries by transovarian route [10]. Additionally, uptake of *Salmonella* by hen’s macrophages after bacterial invasion of intestinal cells aids in its dissemination within the host, including the reproductive organs [11–14]. Contamination of egg contents (yolk, albumen and eggshell membranes) by SE can occur before oviposition [11,12], where *Salmonella* colonized in reproductive organs invades and multiplies in the granulosa cells of the preovulatory follicles in the reproductive tract [15,16]. Since SE colonization in the ceca of layers results in the systemic spread of the pathogen to reproductive organs by transovarian route, decreasing the pathogen prevalence in flocks has been reported to result in a direct reduction in human health risk [17]. Control measures implemented at the flock level could reduce human salmonellosis from egg consumption, and was thus suggested as a primary focus of control at farm level [4]. Therefore, innovative on-farm strategies for preventing colonization of birds with SE are critical to prevent the pathogen contamination of eggs. Apart from reducing the colonization of SE in chicken cecum, a viable approach could be one that potentially reduces the bacterial virulence mechanisms, thereby reducing their colonization in the reproductive tract of birds, where eggs are formed [10,18,19].

Historically, plants have served as a useful resource for the development of novel drugs against human and animal diseases. Plants produce a wide array of compounds, most of which as a defense mechanism against predation by pathogenic microorganisms and insects. Several plant compounds form dietary constituents as well as active components in a number of herbal and traditional medicines [20]. In recent years, the use of natural compounds has gained attention due to increasing concerns over the safety of synthetic chemicals [21,22] and emerging antibiotic resistance in bacteria [22]. The antimicrobial properties of several plant-derived essential oils have been previously reported, and a variety of active ingredients have been identified [23–25]. Among the various plant-derived antimicrobials (PDAs), Carvacrol (CR) and thymol (THY) are present in oregano oil, which is obtained from *Origanum glandulosum* Desf, whereas eugenol (EUG) is a component of clove oil (*Eugenia caryophillis* Spreng). All the aforementioned PDAs are reported to have a wide spectrum of antimicrobial activity against several Gram-negative and Gram-positive bacteria [26–28], and are classified as generally recognized as safe (GRAS) for addition in food products by the FDA [29–32]. Our laboratory previously reported that *Trans*-cinnamaldehyde (TC) and EUG were effective in killing SE *in vitro* [33,34] and reduced SE colonization in broiler chickens *in vivo* [35]. Additionally, research conducted in our laboratory revealed that sub-inhibitory concentrations (SIC, concentration not inhibiting bacterial growth) of TC reduced the attaching and invading abilities of uropathogenic *Escherichia coli* on human urinary tract epithelial cells, by down-regulating the expression of genes critical for host tissue colonization [36]. The current study was undertaken to investigate the efficacy of SICs of CR, THY and EUG in reducing the attachment to and invasion of primary chicken oviduct epithelial cells (COEC) by SE *in vitro*. Moreover, the effect of CR, THY and EUG on the transcription of various virulence genes critical for SE colonization in the chicken oviduct was studied.

## 2. Results and Discussion

*S.* Enteritidis is a major cause of food-borne disease worldwide, with the consumption of contaminated egg as the common source of human infection. In chickens, there are two possible ways by which the pathogen contaminates the eggs; by directly contaminating the outer surface of the eggs while transiting the cloaca, or by reaching the ovarian tissue *via* systemic circulation and contaminating the yolk prior to oviposition [15]. Salmonellae have been found on the mucosal surface and within the epithelial cells lining the oviduct in naturally and experimentally infected hens [37,38]. Since the attachment and invasion of SE in chicken oviduct cells are essential steps in its colonization of ovarian tissue and contamination of egg yolk [10,13,39], a potential strategy for controlling trans ovarian transmission of SE to eggs is to reduce pathogen colonization in the ovarian tissue, thereby decreasing its entry into the eggs. Therefore, this study investigated the efficacy of PDAs in reducing SE adhesion to and invasion of COEC.

Although all parts of chicken reproductive tract are prone to SE colonization, the isthmus is a critical site in terms of persistent reproductive tract colonization and egg membrane contamination by the pathogen before deposition of eggshell membranes [40,41]. Since no established chicken oviduct epithelial cell lines are available commercially, we isolated primary COEC from the isthmus of chicken oviduct. The primary COEC model has been reported as a useful tool in studying the early interactions between SE and chicken oviduct epithelium [39,42,43]. Several studies have suggested that the epithelial cells (along with the lymphocytes and macrophages associated with the epithelial cells) of laying hens express a number of beta-defensins (AvβDs), which are antimicrobial peptides that play significant roles in the innate immune systems of the chickens [44–46]. Hence, in order to validate the COEC isolated in the current study, the presence of six AvβD genes was detected using RT-qPCR [42,47,48]. Our results indicated that the oviduct epithelial cells derived from laying hens constitutively expressed all AvβD genes (data not shown), thereby confirming that the cell line used in the study was of chicken oviduct origin.

**Figure 1:**
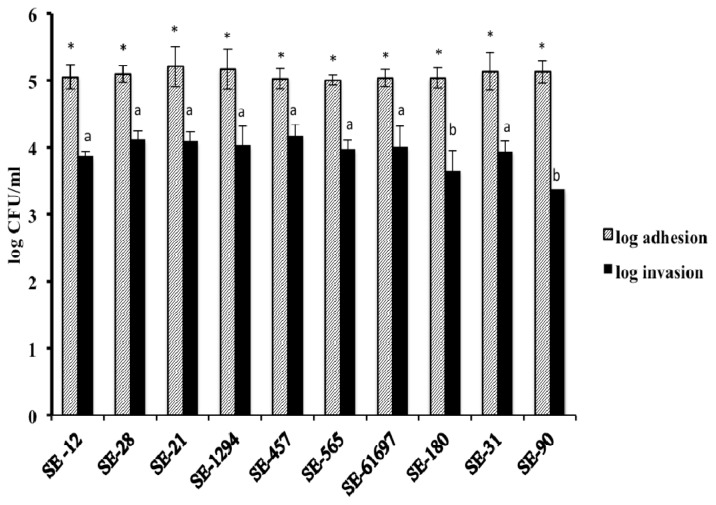
Adhesion to and invasion of 10 strains of *Salmonella* Enteritidis (SE) on chicken oviduct epithelial cells (COEC). Ten strains of SE were screened to determine their adhesive and invasive properties on COEC. The COEC were seeded on a 24-well tissue culture plates at ~10^5^ cells per well, and inoculated with ~6.0 log CFU of each SE (MOI 10). The infected monolayer was incubated for 1 h following which the cells were lysed and the number of viable adherent SE was determined. For the invasion assay, the monolayers incubated for 1 h following SE infection, were rinsed and incubated for another 2 h in whole media-10% FBS containing gentamicin (100 μg/mL) following which the cells were lysed and SE was enumerated. * Striped bars with asterisks representing adhesion of isolates on COEC did not differ significantly from each other (*p* > 0.05). ^a,b^ Black bars with different superscripts differed significantly in invasion of isolates to COEC (*p* < 0.05).

Figure 1 shows the adhesion to and invasion of 10 SE isolates on the COEC. There was no significant difference (*p* > 0.05) between the adhesion and invasion capabilities of the isolates on the primary cell line except for the invasion of SE 180 and SE 90 (*p <* 0.05). On average, ~5.0 log and 4.0 log CFU of SE attached and invaded the COEC, respectively. The mean attachment and invasion efficiencies of the 10 SE isolates on COEC were 85% and 65%, respectively. This is in accordance with a previous study, wherein a similar adhesion and invasion efficiency of SE on COEC was observed [49]. From the 10 isolates, three strains, namely SE 21, SE 28 and SE 457 were chosen for the subsequent experiments.

The two SICs of the PDAs that did not inhibit SE growth as compared to control were selected, which included 0.50 and 0.60 mM for CR and THY, and 1.2 and 1.8 mM for EUG. The average initial SE population in the control and treated samples was ~6.0 log CFU/mL. Following incubation at 37 °C for 24 h, ~8.0 log CFU/mL of SE was recovered from all wells, irrespective of control and compound treatment (data not shown). This confirmed that the concentrations used in the assay were not inhibitory on the bacterium.

**Figure 2:**
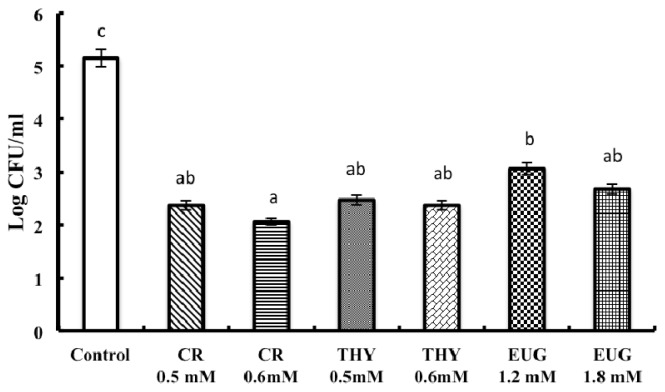
Effect of carvacrol (CR) thymol (THY) and eugenol (EUG) on SE adhesion to primary COEC. COEC (10^5^ cells) were inoculated with 6.0 log CFU of each SE (MOI 10). After incubating the infected monolayer for 1 h at 39 °C, the cells were washed three times followed by lysis using triton X and viable SE adhered were enumerated. For invasion, following incubation for 1 h, the infected cells were rinsed and incubated for another 2 h in whole media supplemented with 10% FBS containing gentamicin (100 μg/mL). The cells were lysed and invading SE was determined. Since there was no significant difference between three strains studied, results are shown for SE 28 (*p* < 0.001). Treatments for each compound differed significantly from the control (open column) at *p* < 0.001.

**Figure 3:**
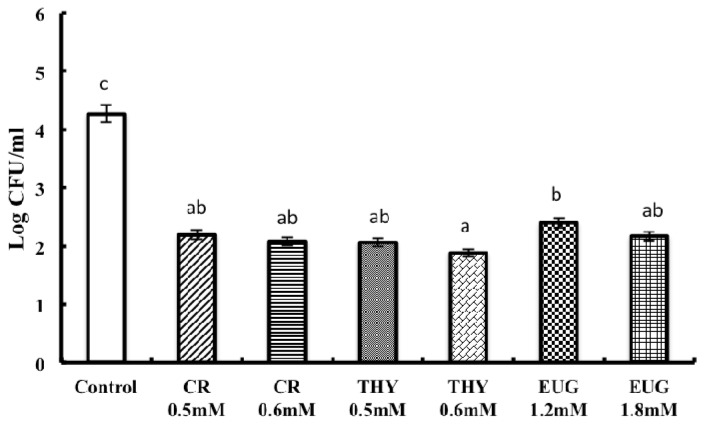
Effect of carvacrol (CR) thymol (THY) and eugenol (EUG) on SE invasion of primary COEC. The COEC were seeded in a 24-well tissue culture plates at ~10^5^ cells per well, and inoculated with ~6.0 log CFU of each SE (MOI 10). The infected monolayer was incubated for 1 h at 39 °C. The cells were washed thrice with PBS followed by triton mediated cell lysis and the number of viable adherent SE was enumerated. For the invasion assay, the monolayers incubated for 1 h following SE infection, were rinsed with minimal media and incubated for additional 2 h in whole media-10% FBS containing gentamicin (100 μg/mL). Following the incubation, the cells were lysed and invading SE was enumerated. Since there was no significant difference between three strains studied, results are shown for SE 28 (*p* < 0.001). Treatments for each compound differed significantly from the control (open column) at *p* < 0.001.

All three PDAs significantly reduced SE adhesion and invasion of COEC (*p* < 0.001) (Figures 2 and 3). In general, the PDAs decreased SE adhesion and invasion of COEC by ~3 log CFU/mL and 2.5 log CFU/mL respectively. It was also observed that all PDAs at their higher tested SIC were more inhibitory on SE adhesion and invasion of COEC compared to the lower SIC (*p* < 0.001). Since there was no significant difference observed between the 3 selected strains, only the data for strain 28 is represented in Figures 2 and 3.

Since SICs of antimicrobials, including antibiotics can modulate bacterial physio-chemical functions, including that of genes, they are used for studying the effect of antimicrobials on bacterial gene expression and virulence [50,51]. Moreover, since the SICs do not inhibit bacterial growth or reduce their populations, the reduction in SE adhesion and invasion of COEC may be due to the effect of PDAs in modulating the expression of genes associated with virulence in the bacterium. To ascertain this, we determined the effect of PDAs on transcription of 22 published genes critical for colonization of chicken reproductive tract by RT-qPCR (Table 4). The RT-qPCR results indicated that all three PDAs significantly down-regulated (*p* < 0.001) several oviduct-specific colonization genes in SE (Table 1). The down-regulated genes included those critical for regulating *Salmonella* motility, namely *flgG* [14], *fimD* [37,52], and *prot6E* [19]; adherence and invasion, *sopB* [49], and *invH* [53]; type three secretion system (TTSS) genes, *sipA*, *sipB*, *pipB*, *ssaV*, and *orf245* [49]; cell membrane and cell wall integrity, *hflK*, *lrp*, *ompR*, and *tatA*, [14]; exo/endonuclease activity, *xthA* [54] and *mrr1/SEN4287* [19] and those involved in metabolism such as *rfbH* [55], *rpoS*, [56], and *ssrA* [57]. Among these genes, *ssaV* and *pipB*, although are integral of *Salmonella* TTSS, they also play a major role in macrophage survival of SE in the host cells [49]. In addition, *ssrA* has been observed to be associated with *Salmonella* survival in macrophages [57]. Other genes reported to play a role in *Salmonella* survival in macrophages are *sodC* [58], *spvB* [59,60] and *mgtC* [61]. The gene *spvB* ribosylates actin of the macrophages and destabilizes the cytoskeleton [59,60]. Yet another virulence gene studied, *invH*, is an outer membrane lipoprotein responsible for *Salmonella* adhesion and invasion of the host cell [53], which in turn is facilitated by *sopB*, that allows the uptake of the pathogen into the host system [49]. On the other hand, *orf245* [49] and *prot6E* [19] are specific to oviduct colonization of SE and *pipB*, *sipA* and *sipB* aid *Salmonella* invasion and translocation of proteins through the TTSS [49]. The genes and their respective functions are provided in Table 4. Although all three PDAs significantly down-regulated the expression of the aforementioned virulence genes, THY followed by CR, and EUG were effective in descending order in decreasing the expression of most of the tested genes (Table 1). These results collectively suggest that these PDAs may be acting through different mechanisms, and genome-wide studies are needed to fully understand the mechanisms by which CR, THY and EUG attenuate virulence in SE.

**Figure 4:**
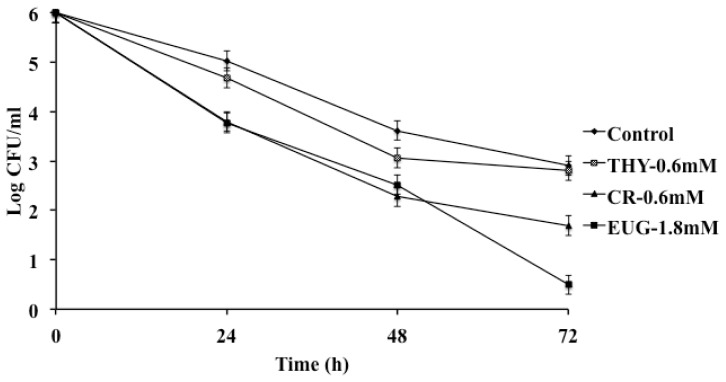
Effect of carvacrol (CR) thymol (THY) and eugenol (EUG) on survival of SE 21 in chicken macrophages. About 10^5^ macrophages were infected with 6.0 log CFU SE, and incubated at 39 °C for 45 min under 5% CO_2_. The macrophages were then washed twice and maintained in whole media supplemented with 10 μg of gentamicin/mL for 72 h. At 24, 48 and 72 h, the cells were lysed and the surviving SE was enumerated on XLD and TSA. All treatments except for THY differed significantly from the control at *p <* 0.001.

**Figure 5:**
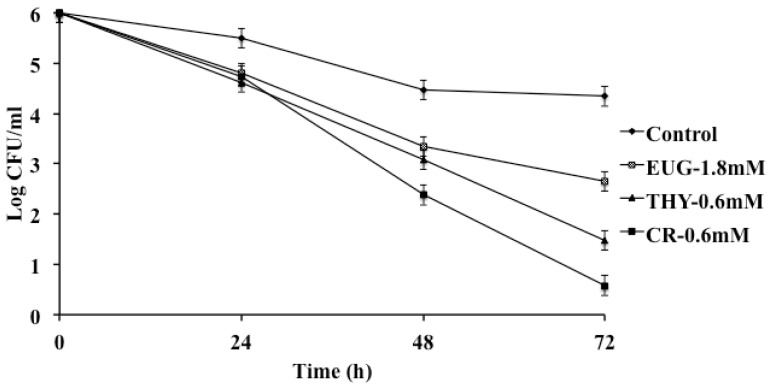
Effect of carvacrol (CR) thymol (THY) and eugenol (EUG) on SE 28 survival of HTC (Chicken macrophages). Macrophages (10^5^ cells) were infected with 6.0 log CFU/mL of SE, and incubated for 45 min, following which they were washed and maintained in whole media supplemented with 10 μg of gentamicin/mL for 24, 48 and 72 h. The cells were lysed at each time point and surviving SE was determined by plating on XLD and TSA. Treatments for each compound differed significantly from the control (black column) at *p* < 0.05.

The uptake of a pathogen by macrophages is a process that helps the host to defend against an invading bacterium and elicit a specific immune response. However, the ability of a pathogen to survive in the hostile environment within the macrophage offers it protection from the immune system and helps in its dissemination [62]. *S*. Enteritidis has the ability to persist in chicken macrophages, enabling its spread via the circulatory system to various internal organs, including the reproductive system [49]. The results from the macrophage survival assay revealed that the PDAs significantly decreased the survival of SE in chicken macrophages, although at different levels (Figures 4 and 5). For example, in Strain 21, except THY, all PDAs decreased SE survival in macrophages by ~1.5 to 2.0 log CFU/mL at 24 and 48 h, and ~2.0 to 3.0 log CFU/mL by 72 h of incubation compared to controls (*p* < 0.001). However, in strain 28 all PDAs, including THY, were found to be effective in reducing SE survivability in macrophages (*p* < 0.001). Since SE 457 failed to survive in macrophages even in the absence of PDAs (control), no data are available for inclusion in the manuscript. RT-qPCR results supported the findings from macrophage survival assay, where the PDAs significantly down-regulated critical genes required for the pathogen survival in the macrophages. We observed that *sodC* was downregulated significantly by PDAs except EUG (Table 1). *sodC*, is a critical gene for *Salmonella* survival in macrophages. Pathogens present in polymorphonuclear cells and macrophages are exposed to reactive oxygen species (ROS) that function to kill bacteria. As a defense mechanism, bacteria up-regulate the expression *sod*, producing the enzyme, superoxide dismutase to neutralize ROS [63]. Similar results were observed on *mgtC* expression, which is required for *Salmonella* growth at low-Mg^2+^ concentrations and intra-macrophage survival [61]. Other genes for macrophage survival, including *ssaV*, *pipB* and *ssrA* were also significantly down regulated by the PDAs (Table 1).

## 3. Experimental Section

### 3.1. Bacterial Strains and Culture Conditions

Ten strains of SE (Table 2) were screened to determine their adhesive and invasive properties on COEC. Since we did not observe any significant variation in the adhesive and invasive abilities of the 10 SE strains on COEC, three strains of SE, namely SE 28 (oviduct isolate), SE 21 (intestinal isolate), and SE 457 (egg yolk isolate) were selected for further investigations. All bacteriological media were purchased from Difco (Becton Dickinson, Sparks, MD, USA). Each strain of SE was cultured separately in 10 mL of sterile tryptic soy broth (TSB) in 50 mL screw-cap tubes, and incubated at 37 °C for 18 h. Following incubation; the cultures were pelleted by centrifugation (3600× *g* for 15 min) at 4 °C, washed twice and resuspended in 10 mL of sterilized phosphate buffered saline (PBS, pH 7.2). Serial, ten-fold dilutions were plated on duplicate tryptic soy agar (TSA) and xylose lysine deoxycholate agar (XLD) agar plates, followed by incubation at 37 °C for 24 h for bacterial enumeration [33,64].

### 3.2. PDAs and SIC Determination

CR, EUG and THY were purchased from Sigma-Aldrich Inc (St. Loius, MO, USA). The SICs of each plant compound were determined as described previously [34–36]. Briefly, sterile 24-well polystyrene tissue culture plates (Costar, Corning Incorporated, Corning, NY, USA) containing 1 mL of TSB were inoculated separately with ~6.0 log CFU of SE, followed by the addition of 1 to 10 μL of CR, THY or EUG in increments of 0.5 μL. The plates were incubated at 37 °C for 24 h, and bacterial growth was determined by plating on TSA and XLD plates. The highest two concentrations of each plant compound below its respective minimum inhibitory concentration that did not inhibit bacterial growth after 24 h of incubation were selected as its SICs for this study. Duplicate samples for each plant compound were included and the experiment was repeated three times.

### 3.3. Isolation of Chicken oviduct Epithelial Cells

Primary COEC were isolated as described previously [42], with slight modifications. The oviduct tissues of 25–28 weeks old, *Salmonella*-free layer hens (single comb, white leghorn) were obtained from the University of Connecticut poultry farm. The isthmal portion of the oviduct was removed, flushed thoroughly with HBSS (Sigma-Aldrich) containing 200 U/mL penicillin (Sigma-Aldrich) and 200 mg/mL streptomycin (Sigma-Aldrich). The epithelial cells were gently scraped of the tissue and treated with 20 mL of HBSS containing 1 mg/mL collagenase (Sigma-Aldrich) for 30 min at 37 °C. After collagenase treatment, the supernatant was discarded and trypsinization of tissue fragments was done using 0.25% trypsin and 3 mM EDTA in 20 mL of HBSS for 10 min at 37 °C. The cell suspension was added with 10% heat-inactivated fetal bovine serum (HI-FBS; Gibco, Invitrogen) to stop the activity of trypsin. The cell suspension was then passed through a cell strainer (100 μm) in order to remove any undigested tissue. The epithelial cells were centrifuged at 50× *g* for 5 min to separate cell aggregates from erythrocytes, platelets, and other immune cells. The supernatant obtained after centrifugation was discarded, and the pellet containing epithelial cells was resuspended in minimal essential medium (MEM, Invitrogen, Grand Island, NY, USA) supplemented with 10% HI-FBS, 2% heat-inactivated chicken serum (HICS; Gibco, Invitrogen), insulin (0.12 U/mL, Sigma-Aldrich), and estradiol (50 nM, Sigma-Aldrich). The COEC were incubated in a tissue culture flask for 2 h at 39 °C in 5% CO_2_ to allow fibroblast attachment. Following incubation, the unattached epithelial cells were collected by gentle pipetting, followed by centrifugation at 125× *g* for 10 min. The pelleted epithelial cells were resuspended in whole medium and allowed to grow until a monolayer was formed. After four successive passages, the cells were seeded onto 24-well cell culture plates (~2 × 10^5^ cells per well), and grown at 39 °C under 5% CO_2_ for 24–36 h. The identity of COEC was confirmed by determining the constitutive expression of avian β-defensin (AvβD) genes (Table 3) by reverse transcriptase quantitative PCR (RT-qPCR) [42].

### 3.4. SE Adhesion and Invasion Assay

The adhesive and invasive abilities of ten *S*. Enteritidis isolates (Table 2) on COEC were investigated [65]. The COEC were seeded on a 24-well tissue culture plates at ~10^5^ cells per well, and inoculated with ~6.0 log CFU of each SE isolate separately (MOI 10). The inoculated COEC cells were incubated at 39 °C in a humidified, 5% CO_2_ incubator. The infected monolayer was incubated for 1 h to facilitate SE attachment, followed by washing to remove unattached bacteria. The cells were then lysed with 0.1% Triton X-100. The number of viable adherent SE was determined by serial dilution and plating on TSA and XLD plates. For the invasion assay, the monolayers were incubated for 1 h following SE infection, rinsed three times in minimal media and incubated for another 2 h in whole media-10% FBS containing gentamicin (100 μg/mL; Sigma-Aldrich) to kill the extracellular bacteria. The wells were then washed with PBS three times, one mL of PBS containing 0.1% Triton X (Invitrogen) was added, followed by incubation at 39 °C for 15 min to lyse the cells and release the invaded SE. The cell lysates were serially diluted, plated on TSA/XLD plates and incubated at 37 °C for 24 h. The assays were run in duplicates and replicated three times.

The effect of CR, THY and EUG on *Salmonella* adhesion to and invasion of COEC was determined as above, except that the bacteria were grown to midlog phase without (control) and with the respective SICs of each plant compound before inoculating onto COEC.

### 3.5. Macrophage Cultivation and SE Survival Assay

Chicken macrophages (HTC, chicken monocyte cell line; [66]) were cultivated in RPMI 1640 with 10% FBS. The cells were activated and plated as described previously [62], with slight modifications. Twenty four hours prior to infection, the cells were seeded on to 24-well tissue culture plates and incubated at 39 °C under 5% CO_2_ to form a monolayer. Each SE isolate grown to midlog phase in the presence or absence of SICs of CR, THY, or EUG was centrifuged (3600× *g*), and resuspended in RPMI media with 10% FBS. About 10^5^ macrophages were separately infected with 6.0 log CFU of each SE isolate at an MOI of 10, and incubated at 39 °C for 45 min under 5% CO_2_. After incubation, the macrophages were treated with whole media containing 100 μg of gentamicin/mL for 2 h at 39 °C to kill extracellular bacteria. The macrophages were then washed twice and maintained in whole media supplemented with 10 μg of gentamicin/mL for 24, 48 and 72 h. The medium was replaced every 24 h. Macrophages were washed twice, lysed with 0.5% Triton X, serially diluted, and plated on TSA and XLD agar plates to determine the surviving population of SE at the aforementioned time intervals. All assays were performed in duplicates at least three times.

### 3.6. RNA Isolation and RT-qPCR

To determine the basal level expression of avian β-defensin (AvβD) genes, RT-qPCR was performed [42] using total RNA extracted from COEC, and primers specific for the AvβD genes (Table 3). Specific amplification of AvβD genes, including AvβD -4, AvβD -5, AvβD -9, AvβD -10, AvβD -11, and AvβD -12 was achieved with primers specific for each gene, and β*-*actin gene serving as the endogenous control. In addition, the effect of CR, THY and EUG on the expression of SE virulence genes was investigated using RT-qPCR. Each SE strain was grown separately with and without the respective SICs of PDAs in TSB at 37 °C to mid-log phase, and total RNA was extracted using RNeasy RNA isolation kit (Qiagen, Valencia, CA, USA). Complementary DNA (cDNA) was synthesized using the Superscript II Reverse transcriptase kit (Invitrogen, Carlsbad, CA, USA), and was used as the template for RT-qPCR. The primers for each gene (Table 4) were designed from published GenBank, SE sequences using Primer Express® software (Applied Biosystems, Foster, CA, USA). Relative gene expression was determined according to comparative critical threshold (Ct) method using a 7500 Fast Real-Time PCR system (Applied Biosystems). Data were normalized to the endogenous control (16S rRNA), and the level of candidate gene expression between treated and control samples were determined.

### 3.7. Statistical Analysis

Data from the adhesion, invasion, and macrophage survival assays were analyzed separately. Completely randomized design (CRD) with factorial treatment structure was followed for all the trials with factors including three plant molecules (CR, EUG, THY), two concentrations and three bacterial strains (21, 28, 457). Each experiment was replicated six times (*n* = 6). Data were analyzed using the mixed procedure of SAS (version 9.3, SAS institute, Inc., Cary, NC, USA) for all assays, and with the repeated measures statement used for the macrophage survival assay (measurements taken at 24, 48 and 72 h of incubation). Least-squares means were generated for significant *F* tests (*p* < 0.001) and separated using least significant differences. For gene expression assays, differences between independent treatments were analyzed using two-tailed *t* tests, and a *p* value of < 0.001 was considered significantly different.

## 4. Conclusions

In conclusion, we found that three PDAs, CR, THY, and EUG significantly reduced SE colonization of cultured chicken oviduct epithelial cells by down-regulating the transcription of critical virulence genes in the bacterium. In addition, the PDAs decreased the survival of SE in chicken macrophages. Further experiments to validate these results in layer chickens are to be conducted.

## Figures and Tables

**Table 1:** Expression of SE genes critical for virulence and oviduct colonization in the presence of CR, THY and EUG *.

Gene	CR 0.50 mM	CR 0.60 mM	THY 0.50 mM	THY 0.60 mM	EUG 1.2 mM	EUG 1.8 mM
*fimD*	−1.88	−1.95	−3.04	−3.67	−6.11	−6.87
*flgG*	−1.81	−2.19	−1.13	−1.24	−3.55	−3.83
*hflK*	−1.40	−2.44	−0.30	−1.90	−3.18	−3.32
*invH*	−16.39	−17.78	−28.55	−29.76	−18.89	−19.81
*lrpF*	−10.05	−13.15	−41.66	−47.61	−1.08	−1.14
*mrr1*	−4.98	−6.22	−8.20	−10.88	−23.95	−24.13
*ompR*	−3.10	−3.66	−5.75	−6.13	−0.15	−0.18
*orf245*	−21.39	−25.37	−60.32	−64.81	−73.29	−76.92
*pipB*	−15.34	−19.16	−65.48	−66.90	−54.38	−54.59
*prot6E*	−1.99	−3.25	−5.99	−8.65	−7.11	−7.92
*rfbH*	−10.41	−11.11	−12.09	−16.66	−1.17	−1.20
*rpoS*	−4.20	−6.89	−3.33	−5.23	−1.33	−1.48
*sipA*	−29.66	−35.24	−78.59	−88.86	−76.30	−77.14
*sipB*	−7.12	−8.33	−14.50	−17.48	−50.29	−53.42
*sodC*	−4.14	−4.20	−8.67	−9.25	−0.22	−0.27
*spvB*	−0.28	−0.44	−0.14	−0.21	−0.12	−0.19
*mgtC*	−4.09	−6.99	−4.52	−7.69	−0.12	−0.23
*sopB*	−22.49	−24.10	−43.22	−46.60	−48.58	−49.25
*ssaV*	−5.05	−6.93	−0.10	−0.10	−1.07	−1.27
*ssrA*	−5.97	−7.04	−3.29	−4.11	0.09	−0.11
*tatA*	−0.61	−1.74	−0.78	−2.08	−0.94	−1.15
*xthA*	−5.02	−5.74	−8.01	−8.26	−0.19	−0.27

* Fold change in gene expression of treatments relative to control (0 mM PDA).

**Table 2:** List of *S.* Enteritidis strains tested for adhesion and invasion capability on COEC.

Strains of SE	Source
SE-21	Chicken intestine isolate (Connecticut Veterinary Diagnostic Medical Laboratory)
SE-28	Chicken oviduct isolate (Connecticut Veterinary Diagnostic Medical Laboratory)
SE-12	Chicken liver isolate (Connecticut Veterinary Diagnostic Medical Laboratory)
SE-31	Chicken gut isolate (Connecticut Veterinary Diagnostic Medical Laboratory)
SE-457	Chicken egg yolk isolate (University of Pennsylvania)
SE-1294	Human egg outbreak (New York Department of Health)
SE-565	Food outbreak (Lunch–II)
SE-61697	Human isolate (University of Pennsylvania)
SE-180	Human isolate (New York Department of Health)
SE-90	Human isolate (Connecticut Veterinary Diagnostic Medical Laboratory)

**Table 3:** List of primers used for RT-qPCR to validate the primary COEC line [38].

Gene	Sequence
β-Actin-F	5′-TGCGTGACATCAAGGAGAAG-3′
β-Actin-R	5′-GACCATCAGGGAGTTCATAGC-3′
AvBD-4-F	5′-CATCTCAGTGTCGTTTCTCTGC-3′
AvBD-4-R	5′-CGCGATATCCACATTGCATG-3′
AvBD-5-F	5′-CTGCCAGCAAGAAAGGAACCTG-3′
AvBD-5-R	5′-GTAATCCTCGAGCAAGGGACA-3′
AvBD-9-F	5′-GCAAAGGCTATTCCACAGCAG-3′
AvBD-9-R	5′-GGAGCACGGCATGCAACAA-3′
AvBD-10-F	5′-TGGGGCACGCAGTCCACAAC-3′
AvBD-10-R	5′-CATGCCCCAGCACGGCAGAA-3′
AvBD-11-F	5′-ACTGCATCCGTTCCAAAGTCTG-3′
AvBD-11-R	5′-GTCCCAGCTGTTCTTCCAG-3′
AvBD-12-F	5′-CCCAGCAGGACCAAAGCAATG-3′
AvBD-12-R	5′-AGTACTTAGCCAGGTATTCC-3′

**Table 4:** List of primers used for RT-qPCR of SE genes and their function.

Accession Number	Gene	Gene Function	Sequence (5′-3′)
NC_011294.1	*fimDF* *fimDR*	Outer membrane usher protein FimD	5′CGCGGCGAAAGTTATTTCAA 3′ 5′CCACGGACGCGGTATCC 3′
NC_011294.1	*flgGF* *flgGR*	Flagellar basal body rod protein	5′GCGCCGGACGATTGC 3′ 5′CCGGGCTGGAAAGCATT 3′
NC_011294.1	*hflKF* *hflKR*	FtsH protease regulator	5′AGCGCGGCGTTGTGA 3′ 5′TCAGACCTGGCTCTACCAGATG 3′
NC_011294.1	*invHF* *invHR*	Cell adherence/invasion protein	5′ CCCTTCCTCCGTGAGCAAA 3′ 5′TGGCCAGTTGCTCTTTCTGA 3′
NC_011294.1	*lrpF* *lrpR*	Leucine-responsive transcriptional regulator	5′TTAATGCCGCCGTGCAA 3′ 5′GCCGGAAACCAAATGACACT 3′
NC_011294.1	*mrr1F* *mrr1R*	Pseudo/restriction endonuclease gene	5′CCATCGCTTCCAGCAACTG 3′ 5′TCTCTACCATGAACCCGTACAAATT 3′
NC_011294.1	*ompRF* *ompRR*	Osmolarity response regulator	5′TGTGCCGGATCTTCTTCCA 3′ 5′CTCCATCGACGTCCAGATCTC 3′
NC_011294.1	*orf245F* *orf245R*	Pathogenicity island protein	5′CAGGGTAATATCGATGTGGACTACA 3′ 5′GCGGTATGTGGAAAACGAGTTT 3′
NC_011294.1	*pipBF* *pipBR*	Pathogenicity island protein	5′GCTCCTGTTAATGATTTCGCTAAAG3′ 5′GCTCAGACTTAACTGACACCAAACTAA 3′
NC_011294.1	*prot6EF* *prot6ER*	Fimbrial biosynthesis	5′GAACGTTTGGCTGCCTATGG 3′ 5′CGCAGTGACTGGCATCAAGA 3′
NC_011294.1	*rfbHF* *rfbHR*	DehydrataseRfbH	5′ACGGTCGGTATTTGTCAACTCA 3′ 5′TCGCCAACCGTATTTTGCTAA 3′
NC_011294.1	*rpoSF* *rpoSR*	RNA polymerase sigma factor RpoS	5′TTTTTCATCGGCCAGGATGT 3′ 5′CGCTGGGCGGTGATTC 3′
NC_011294.1	*sipAF* *sipAR*	Pathogenicity island 1 effector protein	5′CAGGGAACGGTGTGGAGGTA 3′ 5′AGACGTTTTTGGGTGTGATACGT 3′
NC_011294.1	*sipBF* *sipBR*	Pathogenicity island 1 effector protein	5′GCCACTGCTGAATCTGATCCA 3′ 5′CGAGGCGCTTGCTGATTT 3′
NC_011294.1	*sodCF* *sodCR*	Superoxide dismutase	5′CACATGGATCATGAGCGCTTT 3′ 5′CTGCGCCGCGTCTGA3′
NC_011294.1	*sopBF* *sopBR*	Cell invasion protein	5′GCGTCAATTTCATGGGCTAAC 3′ 5′GGCGGCGAACCCTATAAACT 3′
NC_011294.1	*ssaVF* *ssaVR*	Secretion system apparatus protein SsaV	5′GCGCGATACGGACATATTCTG 3′ 5′TGGGCGCCACGTGAA3′
NC_011294.1	*ssrAF* *ssrAR*	Sensor Kinase	5′CGAGTATGGCTGGATCAAAACA 3′ 5′TGTACGTATTTTTTGCGGGATGT 3′
NC_011294.1	*tatAF* *tatAR*	Twin arginine translocase protein A	5′AGTATTTGGCAGTTGTTGATTGTTG 3′ 5′ACCGATGGAACCGAGTTTTTT 3′
NC_011294.1	*xthAF* *xthAR*	Exonuclease III	5′CGCCCGTCCCCATCA 3′ 5′CACATCGGGCTGGTGTTTT 3′
NC_011294.1	*16S f* *16S r*	SENr010, 16S ribosomal RNA	5′CCAGGGCTACACACGTGCTA 3′ 5′TCTCGCGAGGTCGCTTCT 3′
NC_011294.1	*mgtCF* *mgtCR*	Mg (2+) transport ATPase protein C	5′CGAACCTCGCTTTCATCTTCTT 3′ 5′CCGCCGAGGGAGAAAAAC 3′
NC_019120.1	*spvBF* *spvBR*	Actin ADP ribosyltransferase 2C toxin SpvB	5′TGGGTGGGCAACAGCAA 3′ 5′GCAGGATGCCGTTACTGTCA 3′

## References

[1] United States Department of Agriculture http://www.ers.usda.gov/topics/animal-products/poultry-eggs/background.aspx.

[2] Latimer H.K., Jaykus L.A., Morales R.A., Cowen P., Crawford-Brown D. (2002). Sensitivity analysis of *Salmonella* Enteritidis levels in contaminated shell eggs using a biphasic growth model. Int. J. Food Microbiol.

[3] Bialka K.L., Demirci A., Knabel S.J., Patterson P.H., Puri V.M. (2004). Efficacy of electrolyzed oxidizing water for the microbial safety and quality of eggs. Poult. Sci.

[4] Namata H., Méroc E., Aerts M., Faes C., Abrahantes J.C., Imberechts H., Mintiens K. (2008). *Salmonella* in Belgian laying hens: An identification of risk factors. Prev. Vet. Med.

[5] Thomas M.E., Klinkenberg D., Ejeta G., van Knapen F., Bergwerff A.A., Stegeman J.A., Bouma A. (2009). Quantification of horizontal transmission of *Salmonella* Enterica serovar Enteritidis bacteria in pair-housed groups of laying hens. Appl. Environ. Microbiol.

[6] De Vylder J., van Hoorebeke S., Ducatelle R., Pasmans F., Haesebrouck F., Dewulf J., van Immerseel F. (2009). Effect of the housing system on shedding and colonization of gut and internal organs of laying hens with *Salmonella* Enteritidis. Poult. Sci.

[7] (2009). FDA Improves Egg Safety.

[8] Allen-Vercoe E., Woodward M.J. (1999). Colonization of the chicken caecum by afimbriate and aflagellate derivatives of *Salmonella* Enterica serotype Enteritidis. Vet. Microbiol.

[9] Stern N.J. (2008). *Salmonella* species and *Campylobacter jejuni* cecal colonization model in broilers. Poult. Sci.

[10] Gantois I., Ducatelle R., Pasmans F., Haesebrouck F., Gast R., Humphrey T.J., van Immerseel F. (2009). Mechanisms of egg contamination by *Salmonella* Enteritidis. FEMS Microbiol. Rev.

[11] Miyamoto T., Baba E., Tanaka T., Sasai K., Fukata T., Arakawa A. (1997). *Salmonella* Enteritidis contamination of eggs from hens inoculated by vaginal, cloacal, and intravenous routes. Avian Dis.

[12] Okamura M., Kamijima Y., Miyamoto T., Tani H., Sasai K., Baba E. (2001). Differences among six *Salmonella* serovars in abilities to colonize reproductive organs and to contaminate eggs in laying hens. Avian Dis.

[13] Gast R.K., Guraya R., Guard-Bouldin J., Holt P.S., Moore R.W. (2007). Colonization of specific regions of the reproductive tract and deposition at different locations inside eggs laid by hens infected with *Salmonella* Enteritidis or *Salmonella* Heidelberg. Avian Dis.

[14] Gantois I., Ducatelle R., Pasmans F., Haesebrouck F., van Immerseel F. (2008). *Salmonella* Enterica serovar Enteritidis genes induced during oviduct colonization and egg contamination in laying hens. Appl. Environ. Microbiol.

[15] Thiagarajan D., Saeed A.M., Asem E.K. (1994). Mechanism of transovarian transmission of *Salmonella* Enteritidis in laying hens. Poult. Sci.

[16] Thiagarajan D., Saeed M., Turek J., Asem E. (1996). In vitro attachment and invasion of chicken ovarian granulosa cells by *Salmonella* Enteritidis phage type 8. Infect. Immun.

[17] Altekruse S., Koehler J., Hickman-Brenner F., Tauxe R.V., Ferris K. (1993). A comparison of *Salmonella* Enteritidis phage types from egg-associated outbreaks and implicated laying flocks. Epidemiol. Infect.

[18] Keller L.H., Benson C.E., Krotec K., Eckroade R.J. (1995). *Salmonella* Enteritidis colonization of the reproductive tract and forming and freshly laid eggs of chickens. Infect Immun.

[19] Clavijo R.I., Loui C., Andersen G.L., Riley L.W., Lu S. (2006). Identification of genes associated with survival of *Salmonella enterica* serovar Enteritidis in chicken egg albumen. Appl. Environ. Microbiol.

[20] Wollenweber E. (1988). Occurrence of flavonoid aglycones in medicinal plants. Prog. Clin. Biol. Res.

[21] Abee T., Krockel L., Hill C. (1995). Bacteriocins: Modes of action and potentials in food preservation and control of food poisoning. Int. J. Food Microbiol.

[22] Salamci E., Kordali S., Kotan R., Cakir A., Kaya Y. (2007). Chemical compositions, antimicrobial and herbicidal effects of essential oils isolated from Turkish *Tanacetum aucheranum* and *Tanacetum chiliophyllum* var. chiliophyllum. Biochem. Syst. Ecol.

[23] Bilgrami K.S., Sinha K.K., Sinha A.K. (1992). Inhibition of aflatoxin production and growth of *Aspergillus flavus* by eugenol and onion and garlic extracts. Indian J Med Res.

[24] Burt S. (2004). Essential oils: Their antibacterial properties and potential applications in foods—A review. Int. J. Food Microbiol.

[25] Holley R.A., Patel D. (2005). Improvement of shelf life and safety of perishable foods by plant essential oils and smoke antimicrobials. Food Microbiol.

[26] Friedman M., Henika P.R., Mandrell R.E. (2002). Bactericidal activities of plant essential oils and some of there isolated constituents against *Campylobacter jejuni*, *Escherichia coli*, *Listeria monocytogenes*, and *Salmonella enterica*. J. Food Prot.

[27] Chun O.K., Kim D.O., Smith N., Schroeder D., Han J.T., Lee C.Y. (2005). Daily consumption of phenolics and total antioxi- dant capacity from fruit and vegetables in the American diet. J. Sci. Food Agric.

[28] Ali S.M., Khan A.A., Ahmed I., Musaddiq M., Ahmed K.S., Polasa H., Venkateswar Rao L., Habibullah C.M., Sechi L.A., Ahmed N. (2005). Antimicrobial activities of Eugenol and Cinnamaldehyde against the human gastric pathogen *Helicobacter pylori*. Ann. Clin. Microbiol. Antimicrob.

[29] Arrebola M.L., Navarro M.C., Jimenez J., Ocana F.A. (1994). Yield and composition of the essential oil of *Thymus serpylloides* subsp. serpylloides. Phytochemistry.

[30] Adams T.B., Cohen S.M., Doull J., Feron V.J., Goodman J.I., Marnett L.J., Munro I.C., Portoghese P.S., Smith R.L., Waddell W.J. (2004). The FEMA GRAS assessment of cinnamyl derivatives used as flavor ingredients. Food Chem. Toxicol.

[31] Adams T.B., Cohen S.M., Doull J., Feron V.J., Goodman J.I., Marnett L.J., Munro I.C., Portoghese P.S., Smith R.L., Waddell W.J. (2005). The FEMA GRAS assessment of hydroxyl- and alkoxy-substituted benzyl derivatives used as flavor ingredients. Food Chem. Toxicol.

[32] Baskaran Y., Periyasamy V., Venkatraman A.C. (2010). Investigation of antioxidant, anti- inflammatory and DNA-protective properties of eugenol in thioacetamide-induced liver injury in rats. Toxicology.

[33] Kollanoor-Johny A., Darre M.J., Hoagland T.A., Schreiber D.T., Donoghue A.M., Donoghue D.J., Venkitanarayanan K. (2008). Antibacterial effect of trans- cinnamaldehyde on *Salmonella* Enteritidis and *Campylobacter jejuni* in chicken drinking water. J. Appl. Poult. Res.

[34] Kollanoor-Johny A., Darre M.J., Donoghue A.M., Donoghue D.J., Venkitanarayanan K. (2010). Antibacterial effect of trans-cinnamaldehyde, eugenol, thymol and carvacrol against *Salmonella* Enteritidis and *Campylobacter jejuni in vitro*. J. Appl. Poult. Res.

[35] Kollanoor-Johny A., Mattson T., Baskaran S.A., Amalaradjou M.A., Babapoor S., March B., Valipe S., Darre M., Hoagland T., Schreiber D. (2012). Reduction of *Salmonella enterica* serovar Enteritidis colonization in 20-day-old broiler chickens by the plant-derived compounds trans-cinnamaldehyde and eugenol. Appl. Environ. Microbiol.

[36] Amalaradjou M.A., Narayanan A., Venkitanarayanan K. (2011). Trans-cinnamaldehyde decreases attachment and invasion of uropathogenic *Escherichia coli* in urinary tract epithelial cells by modulating virulence gene expression. J. Urol.

[37] De Buck J., van Immerseel F., Meulemans G., Haesebrouck F., Ducatelle R. (2003). Adhesion of *Salmonella enterica* serotype Enteritidis isolates to chicken isthmal glandular secretions. Vet. Microbiol.

[38] Hoop R.K., Pospischil A. (1993). Bacteriological, serological, histological and immunohistochemical findings in laying hens with naturally acquired *Salmonella* Enteritidis phage type 4 infection. Vet. Rec.

[39] De Buck J., Pasmans F., van Immerseel F., Haesebrouck F., Ducatelle R. (2004). Tubular glands of the isthmus are the predominant colonization sites of *Salmonella* Enteritidis in the upper oviduct of laying hens. Poult. Sci.

[40] Cox N.A., Berrang M.E., Cason J.A. (2000). *Salmonella* penetration of eggshells and proliferation in broiler hatching eggs-a review. Poult. Sci.

[41] De Buck J., van Immerseel F., Haesebrouck F., Ducatelle R. (2004). Colonization of the chicken reproductive tract and egg contamination by *Salmonella*. J. Appl. Microbiol.

[42] Ebers K.L., Zhang C.Y., Zhang M.Z., Bailey R.H., Zhang S. (2009). Transcriptional profiling avian beta-defensins in chicken oviduct epithelial cells before and after infection with *Salmonella enterica*serovar Enteritidis. BMC Microbiol.

[43] Jung J.G., Park T.S., Kim J.N., Han B.K., Lee S.D., Song G., Han J.Y. (2011). Characterization and application of oviductal epithelial cells in vitro in *Gallus domesticus*. Biol. Reprod.

[44] Harwig S.S., Swiderek K., Kokryakov V.N., Tan L., Lee T.D., Panyutich E.A., Aleshina G.M., Shamova O.V., Lehrer R.I. (1994). Gallinacins: Cysteine- rich antimicrobial peptides of chicken leukocytes. FEBS Lett.

[45] Lynn D.J., Higgs R., Gaines S., Tierney J., James T., Lloyd A.T., Fares M.A., Mulcahy G., O’Farrelly C. (2004). Bioinformatic discovery and initial characterisation of nine novel antimicrobial peptide genes in the chicken. Immunogenetics.

[46] Xiao Y., Hughes A.L., Ando J., Matsuda Y., Cheng J.F., Skinner-Noble D., Zhang G. (2004). A genome-wide screen identifies a single beta-defensin gene cluster in the chicken: Implications for the origin and evolution of mammalian defensins. BMC Genomics.

[47] Michailidis G., Avdi M., Argiriou A. (2012). Transcriptional profiling of antimicrobial peptides avian β-defensins in the chicken ovary during sexual maturation and in response to *Salmonella* Enteritidis infection. Res. Vet. Sci.

[48] Mageed A.M., Isobe N., Yoshimura Y. (2008). Expression of avian beta defensins in the oviduct and effects of lipopolysaccharide on their expression in the vagina of hens. Poult. Sci.

[49] Li S., Zhang Z., Pace L., Lillehoj H., Zhang S. (2009). Functions exerted by the virulence-associated type-three secretion systems during *Salmonella enterica* serovar Enteritidis invasion into and survival within chicken oviduct epithelial cells and macrophages. Avian Pathol.

[50] Goh E.B., Yim G., Tsui W., McClure J., Surette M.G., Davies J. (2002). Transcriptional modulation of bacterial gene expression by subinhibitory concentrations of antibiotics. Proc. Natl. Acad. Sci. USA.

[51] Fonseca A.P., Extremina C., Fonseca A.F., Sousa J.C. (2004). Effect of subinhibitory concentration of piperacillin/tazobactam on *Pseudomonas aeruginosa*. J. Med. Microbiol.

[52] De Buck J., van Immerseel F., Haesebrouck F., Ducatelle R. (2004). Effect of type 1 fimbriae of *Salmonella enterica* serotype Enteritidis on bacteremia and reproductive tract infection in laying hens. Avian Pathol.

[53] Porter S.B., Curtiss R. (1997). Effect of Inv mutations on *Salmonella* virulence and colonization in 1-day-old White Leghorn chicks. Avian Dis.

[54] Lu S., Killoran P.B., Riley L.W. (2003). Association of *Salmonella enterica* serovar Enteritidis *yafD* with resistance to chicken egg albumen. Infect. Immun.

[55] Gantois I., Ducatelle R., Pasmans F., Haesebrouck F., van Immerseel F. (2009). The *Salmonella* Enteritidis lipopolysaccharide biosynthesis gene *rfbH* is required for survival in egg albumen. Zoonoses Public Health.

[56] Shah D.H., Casavant C., Hawley Q., Addwebi T., Call D.R., Guard-Petter J. (2012). *Salmonella* Enteritidis strains from poultry exhibit differential responses to acid stress, oxidative stress, and survival in the egg albumen. Foodborne Pathog. Dis.

[57] Bohez L., Gantois I., Ducatelle R., Pasmans F., Dewulf J., Haesebrouck F., van Immerseel F. (2008). The *Salmonella* Pathogenicity Island 2 regulator *ssrA* promotes reproductive tract but not intestinal colonization in chickens. Vet. Microbiol.

[58] De Groote M.A., Ochsner U.A., Shiloh M.U., Nathan C., McCord J.M., Dinauer M.C., Libby S.J., Vazquez-Torres A., Xu Y., Fang F.C. (1997). Periplasmic superoxide dismutase protects *Salmonella* from products of phagocyte NADPH-oxidase and nitric oxide synthase. Proc. Natl. Acad. Sci. USA.

[59] Lesnick M.L., Reiner N.E., Fierer J., Guiney D.G. (2001). The *Salmonella spvB* virulence gene encodes an enzyme that ADP-ribosylates actin and destabilizes the cytoskeleton of eukaryotic cells. Mol. Microbiol.

[60] Otto H., Tezcan-Merdol D., Girisch R., Haag F., Rhen M., Koch-Nolte F. (2000). The *spvB* gene-product of the Salmonella enterica virulence plasmid is a mono (ADP-ribosyl) transferase. Mol. Microbiol.

[61] Retamal P., Castillo-Ruiz M., Mora G.C. (2009). Characterization of MgtC, a virulence factor of *Salmonella enterica* serovar Typhi. PloS One.

[62] Townsend S.M., Hurrell E., Gonzalez-Gomez I., Lowe J., Frye J.G., Forsythe S., Badger J.L. (2007). *Enterobacter sakazakii* invades brain capillary endothelial cells, persists in human macrophages influencing cytokine secretion and induces severe brain pathology in the neonatal rat. Microbiology.

[63] Erturk H.N. (1999). Responses of Superoxide Dismutases to Oxidative Stress in *Arabidopsis thaliana*. Ph.D. Thesis.

[64] Kollanoor-Johny A., Baskaran S.A., Charles A.S., Amalaradjou M.A., Darre M.J., Khan M.I., Hoagland T.A., Schreiber D.T., Donoghue A.M., Donoghue D.J. (2009). Prophylactic supplementation of caprylic acid in feed reduces *Salmonella enteritidis* colonization in commercial broiler chicks. J. Food Prot.

[65] Moroni O., Kheadr E., Boutin Y., Lacroix C., Fliss I. (2006). Inactivation of adhesion and invasion of food-borne *Listeria monocytogenes* by bacteriocin-producing Bifidobacterium strains of human origin. Appl. Environ. Microbiol.

[66] Kannan L., Rath N.C., Liyanage R., Lay J.O. (2007). Identification and characterization of thymosin beta-4 in chicken macrophages using whole cell MALDI-TOF. Ann. N. Y. Acad. Sci.

